# Nanoscale Thermometry of Plasmonic Structures via Raman Shifts in Copper Phthalocyanine

**DOI:** 10.1021/acs.jpcc.3c01561

**Published:** 2023-05-11

**Authors:** Pan Li, Sven H. C. Askes, Esther del Pino Rosendo, Freek Ariese, Charusheela Ramanan, Elizabeth von Hauff, Andrea Baldi

**Affiliations:** † Department of Physics and Astronomy, Vrije Universiteit Amsterdam, De Boelelaan 1081, 1081 HV Amsterdam, Netherlands; ‡ Max Planck Institute for Polymer Research, Ackermannweg 10, 55128 Mainz, Germany; § Faculty of Electrical and Computer Engineering, Technical University of Dresden, 01062 Dresden, Germany; ∥ Fraunhofer Institute for Organic Electronics, Electron Beam and Plasma Technology (FEP), 01277 Dresden, Germany

## Abstract

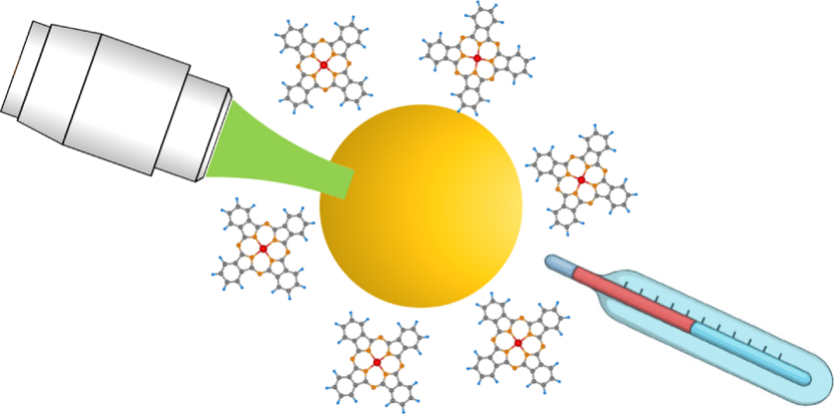

Temperature measurements at the nanoscale are vital for the application of plasmonic structures in medical photothermal therapy and materials science but very challenging to realize in practice. In this work, we exploit a combination of surface-enhanced Raman spectroscopy together with the characteristic temperature dependence of the Raman peak maxima observed in β-phase copper phthalocyanine (β-CuPc) to measure the surface temperature of plasmonic gold nanoparticles under laser irradiation. We begin by measuring the temperature-dependent Raman shifts of the three most prominent modes of β-CuPc films coated on an array of Au nanodisks over a temperature range of 100–500 K. We then use these calibration curves to determine the temperature of an array of Au nanodisks irradiated with varying laser powers. The extracted temperatures agree quantitatively with the ones obtained via numerical modeling of electromagnetic and thermodynamic properties of the irradiated array. Thin films of β-CuPc display low extinction coefficients in the blue-green region of the visible spectrum as well as exceptional thermal stability, allowing a wide temperature range of operation of our Raman thermometer, with minimal optical distortion of the underlying structures. Thanks to the strong thermal response of the Raman shifts in β-CuPc, our work opens the opportunity to investigate photothermal effects at the nanoscale in real time.

## Introduction

Thermal plasmonics is an emerging, active research field that exploits the localized surface plasmon resonance (LSPR) of nanoparticles (NPs) of coinage metals such as Au, Ag, and Cu to generate heat at the nanoscale.^1^ The resonant excitation of metallic NPs leads to the confinement of electromagnetic energy close to their surface and below the diffraction limit of light,^2,3^ resulting in the enhancement of light absorption and scattering in the visible and near-infrared (NIR) regions.^1,4,5^ The light energy is absorbed by the gas of free electrons in metallic NPs, producing a nonequilibrium electronic distribution. Subsequently, the electronic gas relaxes through internal electron–phonon interactions and results in heating of the NP lattice via Joule heating.^3^ This process occurs within several picoseconds.^6−8^ Heat is then transferred from the NP lattice to the surface and then to the environment in a process that typically takes place within nanoseconds,^9^ leading to a temperature gradient extending away from the NP surface. This effect has been exploited in many areas where optically triggered, point-like nanosources of heat can be used, such as photothermal cancer therapy,^10,11^ drug delivery,^12^ microfluidics,^13,14^ and chemical reactions.^15^

Most photothermal applications are based on the use of gold NPs, thanks to their tunable resonance frequency from the visible to the NIR, long-term stability, weak biological toxicity, and easy surface functionalization with different (bio)chemicals.^1,16,17^

The plasmonic conversion of light into heat and the resulting temperature at the surface of the NPs depend on a variety of factors, including the intensity, wavelength, and polarization of the excitation;^18^ the geometry, material, and surface structure of the metal NPs;^19^ and the temperature dependence of the refractive index, heat capacity, and thermal conductivity of the materials involved.^20^ The contribution of all these factors complicates the prediction of photothermal heating effects at the nanoscale and calls for the development of strategies to accurately measure the temperature experimentally at the surface of irradiated plasmonic NPs.

A variety of optical techniques have been developed to determine the local temperature of plasmonic nanostructures, which can be mainly categorized as either (1) near-field thermometry, such as scanning thermal microscopy,^21^ or (2) far-field thermometry, such as refractive index change,^22^ interferometry,^23^ Raman spectroscopy,^24,25^ fluorescence spectroscopy,^26^ and the anti-Stokes emission of the NPs themselves.^27^ These techniques differ in thermal resolution and reliability, as well as suitability for monitoring temperature gradients in different environments and materials. Scanning thermal microscopy can measure the surface temperature of plasmonic NPs with a spatial resolution lower than 100 nm but is invasive due to the proximity of the tip to the sample, which perturbs the local distribution of electromagnetic fields and may impact the surface temperature of the plasmonic nanostructure. On the other hand, far-field thermometry techniques are less invasive. Raman spectroscopy is contactless, nondestructive, and, when coupled to confocal microscopy, is able to identify and analyze chemical substances with high spatial resolution. Raman spectroscopy has been successfully applied for temperature sensing at the nanoscale, by monitoring the intensity ratio of pairs of Stokes and anti-Stokes lines.^25^ Additionally, the intensity of weak Raman signals of molecules closest to the plasmonic NPs may be enhanced by several orders of magnitudes,^17^ allowing detection limits down to the single molecule via surface-enhanced Raman spectroscopy (SERS). The Stokes/anti-Stokes approach to Raman thermometry relies on the ratio of the intensity between these two lines that, in turn, depends on the temperature-dependent population of vibrational states of different modes, according to the Boltzmann distribution. When temperature rises, the anti-Stokes intensity increases due to the progressively larger population of excited vibrational levels, while the Stokes intensity decreases. Despite the elegance of this technique, detecting the weak intensity of low-mode Raman scattering requires expensive (Bragg-type) laser rejection filters, and the Stokes/anti-Stokes intensity ratio can be sensitive to other non-Raman effects such as fluorescence.^28^ Additionally, when combining Stokes/anti-Stokes thermometry with SERS measurements, the selective enhancement of Raman modes from molecules closest to the plasmonic resonance maxima makes it difficult to disentangle the influence of SERS and temperature on the Raman peak intensity.^24,29,30^

Using the Raman peak position to determine nanoscale temperatures avoids many of these drawbacks, as Raman shifts are not influenced by the excitation wavelength, the excitation intensity fluctuations, the refractive index of the medium, or the optical properties of the sample. The thermal response of the Raman peak shifts is related to the change in vibrational frequency of the Raman-active molecules, amplified by the typical anharmonicity of their potential energy surfaces.^31^ Until now, the temperature-dependent Raman shift has been observed in many materials, such as inorganic and organic semiconductors.^24,32−37^

Copper phthalocyanine (CuPc) is a well-studied small-molecule organic semiconducting material. Traditionally, it has been used as an industrial dye or pigment, but over the last decades it has also been studied as an active material in light-emitting diodes,^38^ photovoltaic cells,^39^ and temperature sensors.^40^ As with other phthalocyanine complexes, CuPc has multiple crystalline polymorphs, including the α-, β-, γ-, and χ-polymorphs. The most commonly studied are the α- and β-phases. The α-phase is metastable and has a parallel molecular packing, whereas the β-phase is thermally stable with a herringbone-type molecular packing.^41,42^ The β-phase can be directly deposited by evaporating onto a substrate heated around 240 °C^43,44^ or by annealing an α-CuPc film in a N_2_ atmosphere.^45^

Films of β-CuPc present excellent thermal and optical stability^45,46^ as well as high chemical stability, thanks to their poor solubility in common organic solvents and strong acids or bases.^47^ In addition, β-CuPc films exhibit a transparency window in the blue and green regions of the visible spectrum,^45,48^ making them ideally suited for use in combination with plasmonic gold and silver NPs.

In this work, we exploit the temperature-dependent Raman shifts of β-CuPc films to accurately measure the temperature at the surface of Au nanodisks irradiated with varying laser intensities. We begin by studying the temperature-dependent Raman spectra of β-CuPc films over the temperature range of 100–500 K. We use these data to determine the relationship between the temperature of β-CuPc films and the shift in the position of the major Raman modes. We then apply this relationship to determine the temperature of Au NP arrays as a function of laser power. Finally, we demonstrate that the measured photothermal effects can be quantitatively reproduced, both using analytical expressions and via numerical modeling of the electrodynamic and heat diffusion properties of our samples.

## Experimental Details

### Electron-Beam Lithography Fabrication of the SERS Substrate

Hexamethyldisilazane was used as an adhesion layer for the resist and was spin-coated on a precleaned 24 mm × 24 mm glass substrate to form a monolayer. Then, a 200 nm thick positive resist CSAR 62 (AR-P 6200) from Allresist GmbH and a 60 nm thick anticharging layer of Elektra 92 (AR-PC 5090.2) conductive polymer were subsequently spin-coated on the sample. Next, electron-beam lithography was performed to generate periodic square arrays of nanoholes in the resist employing a Raith e-LiNE lithography system. Samples were exposed to 50 kV accelerating voltage and 1.23 nA beam current. The exposed samples were developed in deionized water, pentyl acetate, *o*-xylene, and a 9:1 mixture of methyl isobutyl ketone and isopropanol at room temperature. Thin films of chromium (Kurt J. Lesker) and gold (IAM Drijfhout) were deposited using conventional thermal evaporation. A ∼5 nm thick chromium layer was used to improve the adhesion of gold on glass, deposited at a rate of ∼0.01 nm/s. The gold layer thickness was 30 nm, deposited at a rate of ∼0.05 nm/s. After evaporation, a lift-off process was used to remove the resist from the substrate and obtain 2 mm × 2 mm areas on the glass substrate covered with periodic square arrays of gold nanodisks with a diameter of 90 nm and an interparticle distance of 160 nm. The quality of the fabricated SERS substrate consisting of the Au nanodisk array was checked by atomic force microscopy (AFM) using a Bruker Veeco Dimension 3100.

### Evaporation of β-CuPc Films

CuPc powder (99.9%) from Sigma-Aldrich was used without further purification prior to deposition and loaded into a tungsten crucible. The CuPc films were grown by thermal evaporation in a vacuum chamber onto both the surface of the sample with Au nanodisks and the bare glass substrate until a desired thickness of 20 nm was reached. The pressure in the vacuum chamber during the evaporation process was kept constant at about 6 × 10^–7^ mbar. The substrate was left at ambient temperature during deposition, and the evaporation rate varied between 0.05 and 0.1 nm/s. Film thickness and deposition rate were monitored by a quartz crystal oscillator coupled to an Inficon SQC-310 deposition controller. To induce the α → β transition, after deposition, the CuPc film was annealed at 300 °C for 3 h in a dry N_2_ atmosphere.

### UV–Vis Spectra, Raman Spectra, and X-ray Diffraction Results

UV–vis spectra were measured using a commercial UV–vis spectrophotometer (Agilent Cary 5000). Raman spectra were obtained using a Renishaw InVia confocal Raman microscope and WIRE software. We used a 532 nm solid-state laser excitation with a 20× magnification microscope objective (*N*_A_ = 0.40) and a laser spot size of 2.46 μm in diameter, as shown in Figure S1. All Raman spectra in this work were acquired using a 1800 lines/mm grating, corresponding to a spectral resolution of 1.6 cm^–1^. The measurements were carried out with the laser light perpendicular to the sample surface in a 180° backscattering configuration. The characteristic Raman band of silicon at 520 cm^–1^ was used to calibrate the spectrometer before each measurement session. The crystal phase of the annealed CuPc film was determined via X-ray diffraction (XRD) using a Bruker D8 diffractometer with Cu Kα (λ = 1.54178 Å) at 40 mA and 40 kV.

### Characterization of β-CuPc Films

UV–vis and vibrational spectroscopies are well-documented tools for identifying crystal-phase transitions and structure distortion in a wide range of materials.^42,48,49^ The α- and β-phases of CuPc films can be distinguished on the basis of relative intensities of the two absorption peaks in the Q-band (see Section S2). The 20 nm thick CuPc film deposited on the SERS substrate was too thin for low-mode Raman and XRD characterizations. To confirm that our thermal treatment was sufficient to convert the as-deposited α-phase to the β-phase, we therefore deposited thicker CuPc films on glass substrates by thermal evaporation and annealed them at 300 °C on a hotplate. A 60 nm thick film was used to discern the α-phase and β-phase from the low-mode and high-mode Raman spectra (see Section S3). A 300 nm thick film was used to characterize the β-phase by XRD (see Section S4).

### Temperature-Dependent Absorbance Spectra

To confirm the thermal stability of the β-CuPc films, we measured the temperature-dependent absorption spectra of a 60 nm thick β-CuPc film at varying temperatures (see Section S5). The measurements were carried out using a setup consisting of a halogen-deuterium lamp (DH2000-DUV, OceanOptics) connected to a USB spectrometer (34000-UV–vis-ES, OceanOptics). The samples were mounted in a continuous flow N_2_ cryostat (Optistat CF-V2, Oxford), equipped with a temperature controller (Mercury iTC, Oxford), and measured under a dynamic vacuum of 10^–4^ to 10^–5^ mbar.

### Calibration of the Raman Thermometer

Temperature-dependent Raman spectra of the Au nanodisk array coated by a 20 nmthick β-CuPc film were acquired with a Microstat HiRes cryostat heating/freezing stage fitted to the microscope stage. The sample was placed on the copper block inside the closed chamber of the cryostat, which was evacuated to a base pressure of ∼6 × 10^–5^ mbar by a turbo-molecular pump to limit convection losses. The laser was focused through the quartz optical window of the cryostat onto the sample. The temperature was controlled by a MercuryiTC temperature controller operated via a LabView software. The sample was cooled with a continuous flow of liquid nitrogen. To reduce perturbation of the sample temperature by heat conduction to the objective and mist generation on the outer surface of the cryostat, dry nitrogen gas was constantly flown between the objective and the top cryostat window. The temperature-dependent Raman spectra were measured at increasing temperatures from 100 to 500 K, waiting 15 min between each temperature to allow the sample to thermally equilibrate. The temperature measurements have an accuracy of 0.5 K. To minimize optical heating during calibration measurements, Raman spectra were collected using a 532 nm cw laser, with a low power of 0.2 mW reaching the sample (see Section S6). The laser power was modulated using a variable neutral density filter. At each temperature, 10 consecutive spectra were collected, each spectrum consisting of 8 accumulations of 10 s. The measured peaks were fitted with Lorentzian functions to determine their Raman shift.

## Results and Discussion

### Sample Design

**Figure 1:**
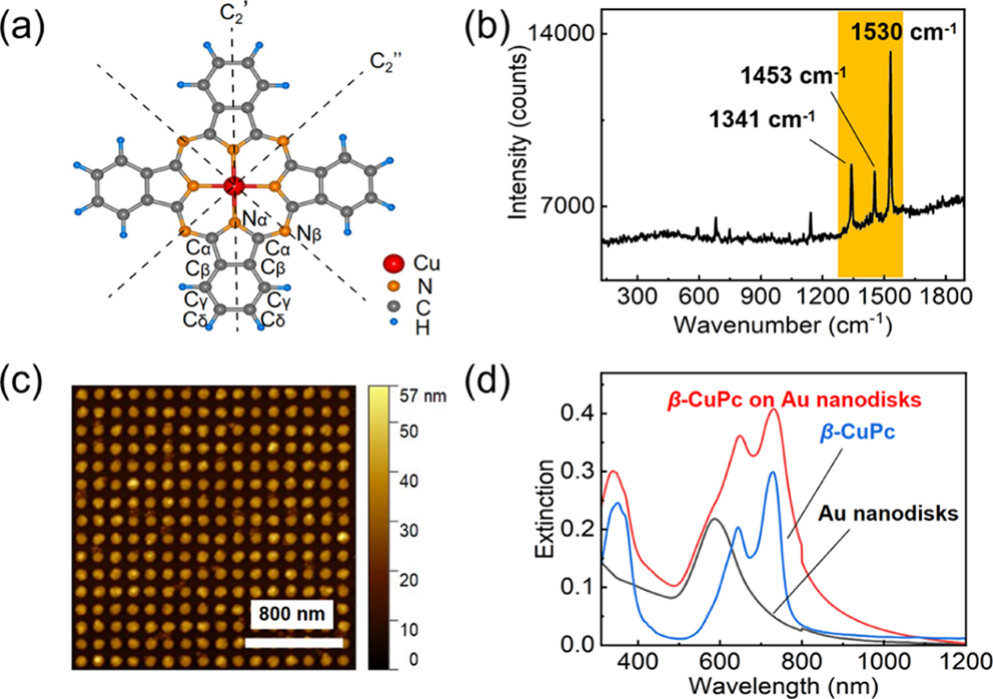
(a) Chemical structure of the CuPc molecule. (b) Room-temperature Raman spectrum of a β-CuPc thin film deposited on an Au nanodisk array. (c) AFM image of an array of Au nanodisks with a diameter of 90 nm, a thickness of 30 nm, and a period of 160 nm. (d) Extinction spectra of (black) a bare Au nanodisk array, (red) an Au nanodisk array coated by a 20 nm thick β-CuPc film, and (blue) a bare β-CuPc film on a glass substrate. The discontinuity at 800 nm is an artifact due to the change of the grating in the spectrometer.

The molecular structure of CuPc is given in Figure 1a. The CuPc molecule, consisting of 57 atoms and possessing *D*_4*h*_ point group symmetry, has a 4-membered phthaloimino group surrounding a central Cu atom. The central symmetric planar structure with an extended π-conjugated system gives rise to its unique optical properties and excellent thermal and chemical stability. As with other highly conjugated molecules, the Raman “fingerprint” of β-CuPc is found in the range 1300–1600 cm^–1^ with three prominent peaks,^50,51^ as shown in Figure 1b. These three peaks have shown a strong spectral dependence on the temperature,^50^ and their assignment, based on experimental and theoretical investigations of single-crystal β-CuPc,^51^ is presented in Table S1 of the Supporting Information.

An AFM image of our Au nanodisk array is shown in Figure 1c. The interparticle distance in our sample is set at ∼160 nm to exclude any near-field coupling. The extinction spectrum of our sample (Figure 1d) is therefore similar to the one predicted for a single Au nanodisk. To maximize the SERS effect and reduce the Lorentzian fitting error of our measured Raman peaks, the LSPR position of the plasmonic substrate should ideally lie between the laser excitation wavelength λ_0_ and the Raman wavelength λ_R_ of interest.^52−54^ In our experiments, we focus on the Raman peaks at 1530, 1453, and 1341 cm^–1^ that, for 532 nm laser excitation, correspond to the wavelengths of 579, 577, and 573 nm, respectively. Our Au nanodisk array exhibits a relatively broad plasmon resonance peak centered around 590 nm and spanning the entire range of interest, hence acting as a good SERS substrate for our experiments. The extinction spectra of the Au nanodisk array coated by a 20 nm thick β-CuPc film and of a bare β-CuPc film on a glass substrate are also shown in Figure 1d.

### Temperature Calibration of the Raman Modes of a β-CuPc Film

**Figure 2:**
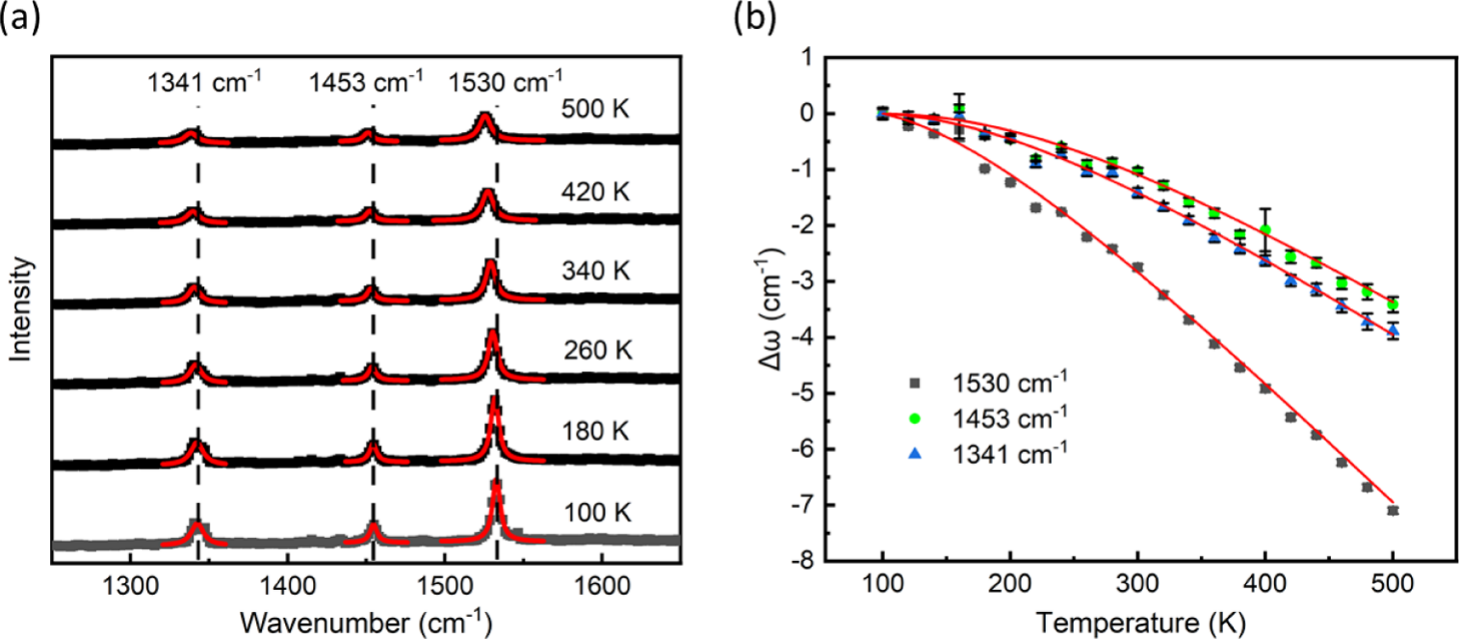
(a) Raman calibration spectra of β-CuPc deposited on Au nanodisks measured at 20 K increments (shown every 80 K for clarity) at temperatures ranging between 100 and 500 K. Each spectrum is the fifth of 10 consecutive acquisitions, each consisting of 80 s integration. The red curves indicate Lorentzian fitting of the individual Raman peaks. (b) Temperature dependence of the change in the Raman shift, Δω = ω(*T*) – ω(100 K), for the three major Raman modes at 1530, 1453, and 1341 cm^–1^. The red lines represent fits to the experimental data using eqs 1 and 2.

We determine the temperature dependence of the three major Raman modes of a 20 nm β-CuPc film deposited on Au nanodisks by measuring the Raman spectra between 100 and 500 K in vacuum (Figure 2). A low laser power of 0.2 mW was used to minimize any direct heating effects. We find that all three Raman modes significantly shift to lower energies (mode softening) at increasing temperatures. At each temperature, the peak position ω(*T*) for the three modes at 1530, 1453, and 1341 cm^–1^ is taken from the average of 10 Raman acquisitions. In Figure 2b, we plot the temperature-dependent change in the Raman peak position, Δω(*T*), defined as the difference between the averaged peak positions at a given temperature and the one at 100 K according to
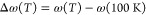
1

The error bars in Figure 2b represent the standard deviation of the peak position for the 10 Raman acquisitions at each temperature. We observe that the three Raman modes display a different temperature-dependent response. The intense Raman peak centered at 1530 cm^–1^ is the most temperature-sensitive, with a maximum shift of up to 7.1 cm^–1^ over a temperature range of 400 K.

The three Raman modes all soften with increased temperature, which can be ascribed to the anharmonicity of the vibrational potential related to thermal expansion of the lattice and phonon–phonon interactions.^33,35,55^ However, many publications have reported that the temperature dependence of Raman frequency can also be described perfectly by only considering phonon–phonon coupling without the thermal expansion.^31,56^ Cui et al. proposed an empirical formula to interpret the temperature dependence of Raman shift in diamond based on phonon–phonon interactions.^31^ Later, the formula was also extended to analyze the temperature dependence of the E_2_ phonon frequency of GaN^57^ and the G band phonon frequency of single-walled carbon nanotubes,^58^ showing good agreement between the theoretical predictions and the experimental results. Using this approach, we can model the temperature dependence of the Raman shift ω(*T*) for the β-CuPc film as
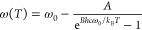
2where ω_0_ is the Raman frequency at 0 K, *k*_B_ is the Boltzmann constant, *h* is the Planck constant, *c* is the speed of light in vacuum in cm/s, *T* is the sample temperature, and *A* and *B* are fitting parameters unique to each vibrational mode and material. The red lines in Figure 2b represent fits to the experimental data using the combination of eqs 1 and 2. The fitting parameters are shown in Table S2 of the Supporting Information.

To verify the sample stability throughout the calibration process, we measured Raman spectra at room temperature before and after the temperature sweep. The two Raman spectra are essentially identical, indicating that the sample does not suffer from structural changes or degradation upon heating and cooling (see Section S7).

### Photothermal Heating of Au NPs

With our calibration at hand, we set out to investigate the photothermal heating induced by laser irradiation of an Au nanodisk array coated with a 20 nm thick β-CuPc layer. In our measurements, a single 532 nm focused laser was used for both optical heating and Raman thermometry. We therefore measured the Raman spectra of our sample at various laser powers in both ascending and random orders. The complete set of peak positions for the β-CuPc/Au nanodisk sample at varying laser powers is presented in Figure S8. The Raman peak position is stable upon repeated measurements under the majority of laser powers, except for some transient shifts at the highest irradiation intensity, most likely due to the buildup of collective photothermal heating effects.

The laser power dependence of the average peak position of all 30 scans is also shown in Figure S8d,h,l. All three peaks show a red shift with increasing excitation power. Such average peak positions, together with the calibration curves of Figure 2b and the fit constants of Table S2, are used to extract the temperature of the array under irradiation, as shown in Figure S9. To ensure that the temperature of the Au nanodisks is unaffected by the weak intrinsic absorption of the β-CuPc overlayer at 532 nm, we also measured the Raman spectra of β-CuPc on bare glass without Au nanodisks at different laser powers (see Figure S10). In the absence of a plasmonic photothermal structure underneath, the spectral positions of the three main Raman peaks were virtually constant for all laser powers tested, confirming that the photothermal contribution of the β-CuPc layer is negligible.

By inverting eqs 1 and 2, the SERS substrate temperature is obtained from the Raman line position ω(*T*) and the fitted parameters ω_0_, *A*, and *B* according to the empirical relationship shown in eq 3 as follows
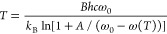
3

**Figure 3:**
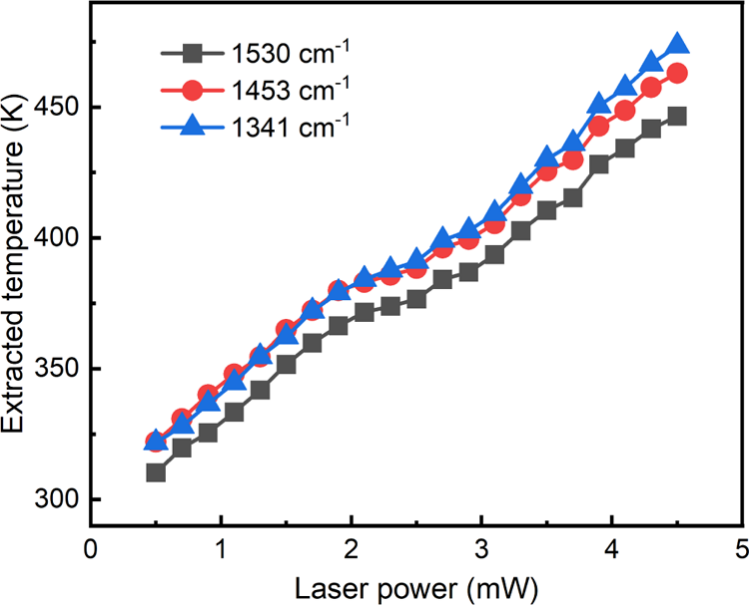
Temperature of the irradiated β-CuPc/Au nanodisk array sample under varying laser powers, extracted from eq 3 for the three major Raman modes. The measurements are conducted at room temperature (296 K).

The temperature of the Au nanodisks as a function of laser power extracted from the three measured peak shifts is shown in Figure 3. The experimentally measured temperature scales linearly with laser power, apart from a slight kink in the data between 375 and 400 K. This effect was systematically observed in three individual data sets, where the laser power was either varied gradually or at random. Based on this observation, we can rule out the random measurement errors, memory, or degradation effects. Furthermore, the CuPc layer had already been annealed at 573 K, which rules out phase transition influences around this temperature range. The physical origin of the temperature-dependent Raman shifts of CuPc is still unknown, and something we are planning to address in a subsequent publication. The slight offset between the three data sets might originate from the error introduced by our calibration procedure, based on fitting the entire data set in the 100–500 K range (Figure 2b) with eq 3.

To verify the accuracy of our Raman thermometer, in the next section, we compare our experimentally derived temperatures against the ones predicted by electrodynamic and heat dissipation numerical models and by analytical expressions.

### Theoretical Prediction by Optical Simulations

**Figure 4:**
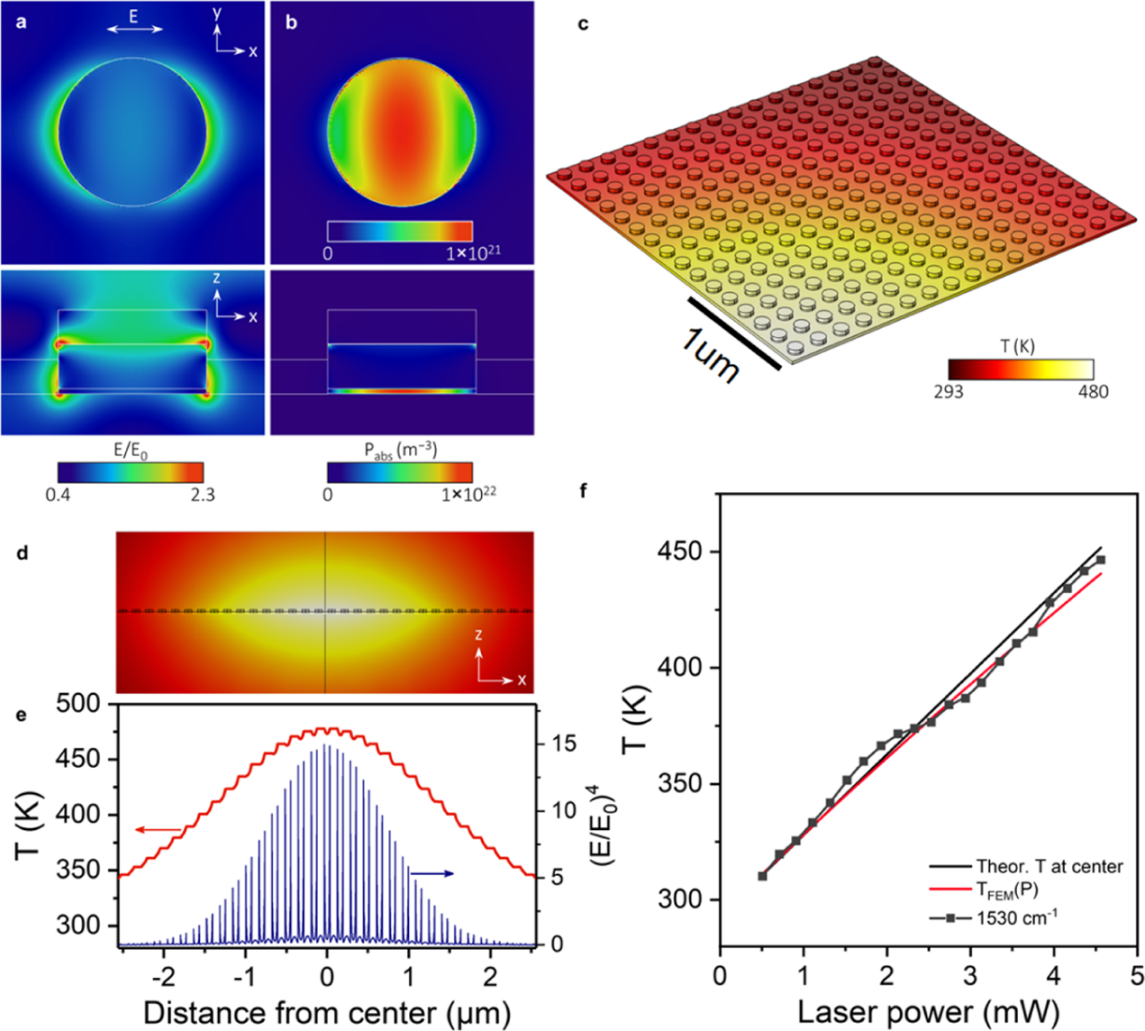
FDTD, FEM, and theoretical modeling of light-to-heat conversion of Au-CuPc NPs. (a,b) FDTD modeling of the electric field (a) and the absorbed optical power (b, unit: m^–3^) of the array unit cell (160 × 160 nm) in the *xy* plane (top, at *z* = 15 nm) and in the *xz* plane (bottom, at *y* = 80 nm, center of the particle). (c) FEM-simulated steady-state surface temperature of the NP array under Gaussian-beam microscope illumination (2.4 μm FWHM) at 532 nm and 4.6 mW optical power. One quarter of the simulated array is shown, with the laser beam located at the bottom corner. (d) *xz* plane temperature crosscut (*x* × *z* = 5.12 × 2 μm) for 4.6 mW optical power for the row of particles closest to the beam center; NPs are outlined in black. (e) Temperature profile at *z* = 15 nm (red) and spatial Raman intensity (*E*/*E*_0_)^4^ (blue) for the row of particles closest to the beam center under 4.6 mW laser. The spikes originate from the dipole resonance of individual NPs (see Figure S13). (f) Experimental (squares), FEM-simulated (red line), and theoretical temperature at the center-most particle (black line) as a function of illumination intensity.

The experimentally determined temperature is not measured at a single point in the array, but it is a convolution of the spatial temperature distribution and the spatial Raman intensity, where the latter is proportional to the electric field inside the CuPc layer to the fourth power (∝*E*^4^, see Figure 4e). To properly reproduce this measured temperature in our model, we numerically calculate the spatial electric-field and optical-heating maps of our sample using finite-difference time domain (FDTD) in combination with heat-transfer finite-element method (FEM) simulations (Figure 4). FDTD simulations were performed for plane-wave excitation of a single nanostructure with periodic boundary conditions (Figure 4a,b) and for Gaussian-beam illumination on a 32 × 32 particle array (∼25 μm^2^, see Supporting Information Figures S11 and S13). As can be seen in Figure 4b, the majority of optical absorption takes place inside the Cr adhesion layer below the Au nanostructure. Figure S13 shows that the electric field is mainly concentrated in the CuPc layer around the nanodisks, with no strong interaction between individual particles. Negligible absorption takes place in the CuPc layer, due to the low absorption coefficient at λ = 532 nm (Figure 1d), which is also confirmed through the laser heating experiments of the bare β-CuPc film, as shown in Figure S10. The Gaussian-beam illumination results mirror these initial observations, and the corresponding absorbed power map (Figure S13) was used as a heat source in FEM heat-transfer simulations (Section S12), yielding spatial heat distribution data (Figure 4c,d).

It was observed that the majority of heating stems from collective heating effects represented as a Gaussian-shaped background (Figure 4e). Finally, to properly compare with the experimentally measured temperature, the obtained temperature distribution was weighted by the Raman intensity distribution within the CuPc domains. Specifically, for each laser intensity, the fraction of Raman intensity at each location within the CuPc layer, *F*_Raman_(*x*,*y*,*z*), was calculated according to
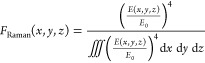
4

This fraction is then multiplied by the temperature distribution, *T*(*x*,*y*,*z*), spatially integrated within the CuPc layer, and divided by the integrated volume of CuPc, *V*_CuPc_
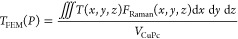
5

The resulting power-dependent temperature curve matched well with the experimentally determined temperatures (Figure 4f), particularly considering that no free fitting parameter was used. The experimental and calculated data agree particularly well for the peak at 1530 cm^–1^, which displays the strongest temperature dependence and is therefore the most reliable peak for thermometry. Minor discrepancies between calculations and experimental data can be attributed to measurement errors in the beam diameter, laser power, absorption, and thermal conductivity of the glass support and CuPc. In conclusion, theoretical and simulation data confirmed that the temperatures measured by our CuPc Raman thermometer are accurate.

### Theoretical Calculations by Analytical Expressions

In steady state, the local temperature of each NP is the sum of ambient temperature, *T*_amb_, heating by the NP itself, Δ*T*_self_, and collective heating by adjacent particles, Δ*T*_coll_^23,59^
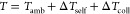
6

Self-heating is proportional to the experimentally determined absorption cross section of each nanodisk (σ_abs_ = 6.3 × 10^–15^ m^2^) and the laser power (*P*, in W), and inversely proportional to the laser beam’s full-width at half-maximum (FWHM) diameter (*H* = 2.4 μm, see Figure S1), the effective thermal conductivity ( = 0.67 W m^–1^ K^–1^), and the NP radius (*r* = 45 nm), according to^23^
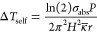
7

For these experimental conditions, self-heating is only ∼6 K at 4.6 mW illumination. On the other hand, for Gaussian-beam illumination, the collective heating contribution at the center of the array is given by^23^
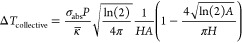
8where *A* is the array unit cell area (2.56 × 10^–14^ m^2^) and amounts to 153 K at 4.6 mW. Thus, in our experimental conditions, heating of the array is dominated by collective effects. The calculated final temperatures of the most central NPs as a function of illumination power are shown in Figure 4f (black line) and correspond well with the derived temperatures from CuPc Raman measurements.

## Conclusions

We demonstrated a temperature sensor based on Raman peak shifts of a β-CuPc thin film with excellent thermal, chemical, and optical stability that is capable of detecting local temperature over a wide temperature range (100–500 K). Even though the Raman laser spot is much larger than the nanodisks, the SERS effect means that the obtained spectra are strongly dominated by those CuPc molecules in the immediate vicinity of the metal. We demonstrate its use by measuring the photothermal effects originating from an array of plasmonic gold nanodisks under varying laser power irradiation. The extracted temperatures fit quantitatively with the ones predicted theoretically. Our demonstration is so far limited to photothermal effects originating from collective heating effects from several tens of NPs. However, we envision that the use of a tailored SERS substrate, such as silver nanodisks of proper aspect ratios, together with a higher spatial resolution of our Raman microscope, could lead to the detection of photothermal effects on single NPs, with potential applications in nanochemistry, nanomedicine, nanolithography, and optoelectronics. The use of such a stable Raman probe molecule as CuPc opens exciting opportunities for the detection of temperature gradients under extreme or otherwise inaccessible conditions. Finally, in this study, we have focused on β-CuPc, thanks to its high thermal and chemical stability and ideal optical properties, but we envision that other thermally responsive and Raman-active materials will be designed and implemented in the future, depending on the specific nanoscale thermometry application.

## Supporting Information Available

Focal spot size of the laser beam; UV–vis spectra, Raman spectra, and XRD patterns of CuPc films; absorption spectra of β-CuPc at varying temperatures; the choice of proper laser power in the calibration process; assignment and symmetry of the Raman peaks of CuPc films; fitting parameters of the data in Figure 2b; sample stability during temperature calibration; time evolution of peak positions at various laser powers; extracted temperature of β-CuPc/Au nanodisks vs laser power; laser power dependence of bare β-CuPc Raman shifts; optical and heat-transfer modeling under Gaussian-beam illumination; heat-transfer model; and the Raman intensity map by simulation (PDF).

## References

[ref1] Baffou G.; Quidant R. Thermo-Plasmonics: Using Metallic Nanostructures as Nano-Sources of Heat: Thermoplasmonics. Laser Photonics Rev. 2013, 7, 171–187. 10.1002/lpor.201200003.

[ref2] Baffou G.; Quidant R. Nanoplasmonics for Chemistry. Chem. Soc. Rev. 2014, 43, 3898 10.1039/c3cs60364d. 24549257

[ref3] Cunha J.; Guo T.; Della Valle G.; Koya A. N.; Proietti Zaccaria R.; Alabastri A. Controlling Light, Heat, and Vibrations in Plasmonics and Phononics. Adv. Opt. Mater. 2020, 8, 2001225 10.1002/adom.202001225.

[ref4] Palermo G.; Cataldi U.; De Sio L.; Bürgi T.; Tabiryan N.; Umeton C. Optical Control of Plasmonic Heating Effects Using Reversible Photo-Alignment of Nematic Liquid Crystals. Appl. Phys. Lett. 2016, 109, 191906 10.1063/1.4967377.

[ref5] Biagioni P.; Huang J.-S.; Hecht B. Nanoantennas for Visible and Infrared Radiation. Rep. Prog. Phys. 2012, 75, 024402 10.1088/0034-4885/75/2/024402. 22790344

[ref6] Askes S. H. C.; Garnett E. C. Ultrafast Thermal Imprinting of Plasmonic Hotspots. Adv. Mater. 2021, 33, 2105192 10.1002/adma.202105192. PMC11468741 34623711

[ref7] Baffou G.; Bordacchini I.; Baldi A.; Quidant R. Simple Experimental Procedures to Distinguish Photothermal from Hot-Carrier Processes in Plasmonics. Light: Sci. Appl. 2020, 9, 108 10.1038/s41377-020-00345-0. 32612818 PMC7321931

[ref8] Wang L.; Zare D.; Chow T. H.; Wang J.; Magnozzi M.; Chergui M. Disentangling Light- and Temperature-Induced Thermal Effects in Colloidal Au Nanoparticles. J. Phys. Chem. C 2022, 126, 3591–3599. 10.1021/acs.jpcc.1c10747. PMC8883463 35242272

[ref9] Chen X.; Chen Y.; Yan M.; Qiu M. Nanosecond Photothermal Effects in Plasmonic Nanostructures. ACS Nano 2012, 6, 2550–2557. 10.1021/nn2050032. 22356648

[ref10] Jaque D.; Martínez Maestro L.; del Rosal B.; Haro-Gonzalez P.; Benayas A.; Plaza J. L.; Martín Rodríguez E.; García Solé J. Nanoparticles for Photothermal Therapies. Nanoscale 2014, 6, 9494–9530. 10.1039/c4nr00708e. 25030381

[ref11] Hirsch L. R.; Stafford R. J.; Bankson J. A.; Sershen S. R.; Rivera B.; Price R. E.; Hazle J. D.; Halas N. J.; West J. L. Nanoshell-Mediated near-Infrared Thermal Therapy of Tumors under Magnetic Resonance Guidance. Proc. Natl. Acad. Sci. U.S.A. 2003, 100, 13549–13554. 10.1073/pnas.2232479100. 14597719 PMC263851

[ref12] Nebu J.; Sony G. Understanding Plasmonic Heat-Triggered Drug Release from Gold Based Nanostructure. J. Drug Delivery Sci. Technol. 2018, 46, 294–301. 10.1016/j.jddst.2018.05.036.

[ref13] Miao X.; Wilson B. K.; Lin L. Y. Localized Surface Plasmon Assisted Microfluidic Mixing. Appl. Phys. Lett. 2008, 92, 124108 10.1063/1.2901192.

[ref14] Liu G. L.; Kim J.; Lu Y.; Lee L. P. Optofluidic Control Using Photothermal Nanoparticles. Nat. Mater. 2006, 5, 27–32. 10.1038/nmat1528. 16362056

[ref15] Kamarudheen R.; Kumari G.; Baldi A. Plasmon-Driven Synthesis of Individual Metal@semiconductor Core@shell Nanoparticles. Nat. Commun. 2020, 11, 3957 10.1038/s41467-020-17789-y. 32770052 PMC7414885

[ref16] Khlebtsov N. G.; Dykman L. A. Optical properties and biomedical applications of plasmonic nanoparticles. J. Quant. Spectrosc. Radiat. Transfer 2010, 111, 1–35. 10.1016/j.jqsrt.2009.07.012.

[ref17] Boisselier E.; Astruc D. Gold Nanoparticles in Nanomedicine: Preparations, Imaging, Diagnostics, Therapies and Toxicity. Chem. Soc. Rev. 2009, 38, 1759 10.1039/b806051g. 19587967

[ref18] Mayer K. M.; Hafner J. H. Localized Surface Plasmon Resonance Sensors. Chem. Rev. 2011, 111, 3828–3857. 10.1021/cr100313v. 21648956

[ref19] Barchiesi D.; Kessentini S.; Guillot N.; de la Chapelle M. L.; Grosges T. Localized surface plasmon resonance in arrays of nano-gold cylinders: inverse problem and propagation of uncertainties. Express 2013, 21, 2245 10.1364/oe.21.002245. 23389205

[ref20] Maier S. A.; Atwater H. A. Plasmonics: Localization and Guiding of Electromagnetic Energy in Metal/Dielectric Structures. J. Appl. Phys. 2005, 98, 011101 10.1063/1.1951057.

[ref21] Desiatov B.; Goykhman I.; Levy U. Direct Temperature Mapping of Nanoscale Plasmonic Devices. Nano Lett. 2014, 14, 648–652. 10.1021/nl403872d. 24422562

[ref22] Baffou G.; Bon P.; Savatier J.; Polleux J.; Zhu M.; Merlin M.; Rigneault H.; Monneret S. Thermal Imaging of Nanostructures by Quantitative Optical Phase Analysis. ACS Nano 2012, 6, 2452–2458. 10.1021/nn2047586. 22305011

[ref23] Baffou G.; Berto P.; Bermúdez Ureña E.; Quidant R.; Monneret S.; Polleux J.; Rigneault H. Photoinduced Heating of Nanoparticle Arrays. ACS Nano 2013, 7, 6478–6488. 10.1021/nn401924n. 23895209

[ref24] Hu S.; Liu B.-J.; Feng J.-M.; Zong C.; Lin K.-Q.; Wang X.; Wu D.-Y.; Ren B. Quantifying Surface Temperature of Thermoplasmonic Nanostructures. J. Am. Chem. Soc. 2018, 140, 13680–13686. 10.1021/jacs.8b06083. 30280886

[ref25] Prezgot D.; Cruikshank J.; Makila-Boivin M.; Birgani S.; Ianoul A. Toward SERS based localized thermometry of Polymer-Supported silver and gold nanostructures. Spectrochim. Acta, Part A 2022, 280, 121514 10.1016/j.saa.2022.121514. 35717928

[ref26] Baffou G.; Kreuzer M. P.; Kulzer F.; Quidant R. Temperature mapping near plasmonic nanostructures using fluorescence polarization anisotropy. Express 2009, 17, 3291 10.1364/oe.17.003291. 19259165

[ref27] Baffou G. Anti-Stokes Thermometry in Nanoplasmonics. ACS Nano 2021, 15, 5785–5792. 10.1021/acsnano.1c01112. 33821619

[ref28] Li L.; Suen B.; Talke F. E. Investigation of Temperature Dependence of Raman Shift of Diamond-Like Carbon Coatings Used in Heat-Assisted Magnetic Recording. IEEE Trans. Magn. 2015, 51, 1–4. 10.1109/tmag.2015.2438298.26203196

[ref29] Pozzi E. A.; Zrimsek A. B.; Lethiec C. M.; Schatz G. C.; Hersam M. C.; Van Duyne R. P. Evaluating Single-Molecule Stokes and Anti-Stokes SERS for Nanoscale Thermometry. J. Phys. Chem. C 2015, 119, 21116–21124. 10.1021/acs.jpcc.5b08054.

[ref30] Itoh T.; Yoshida K.; Tamaru H.; Biju V.; Ishikawa M. Experimental Demonstration of the Electromagnetic Mechanism Underlying Surface Enhanced Raman Scattering Using Single Nanoparticle Spectroscopy. J. Photochem. Photobiol., A 2011, 219, 167–179. 10.1016/j.jphotochem.2011.03.001.

[ref31] Cui J. B.; Amtmann K.; Ristein J.; Ley L. Noncontact Temperature Measurements of Diamond by Raman Scattering Spectroscopy. J. Appl. Phys. 1998, 83, 7929–7933. 10.1063/1.367972.

[ref32] Kim Y.; Chen X.; Wang Z.; Shi J.; Miotkowski I.; Chen Y. P.; Sharma P. A.; Lima Sharma A. L.; Hekmaty M. A.; Jiang Z.; et al. Temperature Dependence of Raman-Active Optical Phonons in Bi_2_Se_3_ and Sb_2_Te_3_. Appl. Phys. Lett. 2012, 100, 071907 10.1063/1.3685465.

[ref33] Huang X.; Gao Y.; Yang T.; Ren W.; Cheng H.-M.; Lai T. Quantitative Analysis of Temperature Dependence of Raman Shift of Monolayer WS_2_. Sci. Rep. 2016, 6, 32236 10.1038/srep32236. 27576751 PMC5006054

[ref34] Kong J. F.; Fan D. H.; Shen W. Z. Anharmonicity Effects in Cu-Doped ZnO Nanocombs by Temperature-Dependent Raman Scattering. Appl. Phys. A: Mater. Sci. Process. 2016, 122, 814 10.1007/s00339-016-0348-5.

[ref35] Burke H. H.; Herman I. P. Temperature Dependence of Raman Scattering in Ge _1–x_ Si_x_ Alloys. Phys. Rev. B: Condens. Matter Mater. Phys. 1993, 48, 15016–15024. 10.1103/physrevb.48.15016. 10008032

[ref36] Alarcón-Lladó E.; Bin-Dolmanan S.; Lin V. K. X.; Teo S. L.; Dadgar A.; Krost A.; Tripathy S. Temperature Rise in InGaN/GaN Vertical Light Emitting Diode on Copper Transferred from Silicon Probed by Raman Scattering. J. Appl. Phys. 2010, 108, 114501 10.1063/1.3505780.

[ref37] Maity S.; Ramanan C.; Ariese F.; MacKenzie R. C. I.; von Hauff E. In Situ Visualization and Quantification of Electrical Self-Heating in Conjugated Polymer Diodes Using Raman Spectroscopy. Adv. Electron. Mater. 2022, 8, 2101208 10.1002/aelm.202101208.

[ref38] Mohan V.; Gautam A. K.; Choudhary S. D.; Bee M. K. M.; Puviarasi R.; Saranya S.; Agrawal N. Enhanced Performance Organic Light Emitting Diode With CuI: CuPC Composite Hole Transport Layer. IEEE Trans. Nanotechnol. 2020, 19, 699–703. 10.1109/tnano.2020.3019096.

[ref39] Ali A. M.; Said D. A.; Khayyat M.; Boustimi M.; Seoudi R. Improving the Efficiency of the Organic Solar Cell (CuPc/C_60_) via PEDOT: PSS as a Photoconductor Layer Doped by Silver Nanoparticles. Results Phys. 2020, 16, 102819 10.1016/j.rinp.2019.102819.

[ref40] Farooq A.; Karimov K. S.; Ahmed N.; Ali T.; Khalid Alamgir M.; Usman M. Copper phthalocyanine and metal free phthalocyanine bulk heterojunction photodetector. Phys. B 2015, 457, 17–21. 10.1016/j.physb.2014.09.032.

[ref41] Hoshino A.; Miyaji H. Redetermination of the Crystal Structure of α-Copper Phthalocyanine Grown on KCl. Acta Crystallogr., Sect. B: Struct. Sci. 2003, 59, 393–403. 10.1107/s010876810300942x. 12761409

[ref42] Zou T.; Wang X.; Ju H.; Zhao L.; Guo T.; Wu W.; Wang H. Controllable Molecular Packing Motif and Overlap Type in Organic Nanomaterials for Advanced Optical Properties. Crystals 2018, 8, 22 10.3390/cryst8010022.

[ref43] Hassan A. K.; Gould R. D. Structural Studies of Thermally Evaporated Thin Films of Copper Phthalocyanine. Phys. Status Solidi A 1992, 132, 91–101. 10.1002/pssa.2211320110.

[ref44] E J.; Kim S.; Lim E.; Lee K.; Cha D.; Friedman B. Effects of substrate temperature on copper(II) phthalocyanine thin films. Appl. Surf. Sci. 2003, 205, 274–279. 10.1016/s0169-4332(02)01115-7.

[ref45] Karan S.; Mallik B. Effects of Annealing on the Morphology and Optical Property of Copper (II) Phthalocyanine Nanostructured Thin Films. Solid State Commun. 2007, 143, 289–294. 10.1016/j.ssc.2007.05.043.

[ref46] Wöhrle D.; Schnurpfeil G.; Makarov S. G.; Kazarin A.; Suvorova O. N. Practical Applications of Phthalocyanines – from Dyes and Pigments to Materials for Optical, Electronic and Photo-Electronic Devices. MHC 2012, 5, 191–202. 10.6060/mhc2012.120990w.

[ref47] Ghani F.; Kristen J.; Riegler H. Solubility Properties of Unsubstituted Metal Phthalocyanines in Different Types of Solvents. J. Chem. Eng. Data 2012, 57, 439–449. 10.1021/je2010215.

[ref48] McAfee T.; Hoffman B. C.; You X.; Atkin J. M.; Ade H.; Dougherty D. B. Morphological, Optical, and Electronic Consequences of Coexisting Crystal Orientations in β-Copper Phthalocyanine Thin Films. J. Phys. Chem. C 2016, 120, 18616–18621. 10.1021/acs.jpcc.6b05043.

[ref49] Tong W. Y.; Chen H. Y.; Djurišić A. B.; Ng A. M. C.; Wang H.; Gwo S.; Chan W. K. Infrared photoluminescence from α- and β-copper phthalocyanine nanostructures. Opt. Mater. 2010, 32, 924–927. 10.1016/j.optmat.2010.01.026.

[ref50] Ghorai U. K.; Mazumder N.; Mamgain H.; Roy R.; Saha S.; Chattopadhyay K. K. Raman Spectroscopic Observation of Gradual Polymorphic Transition and Phonon Modes in CuPc Nanorod. J. Phys. Chem. C 2017, 121, 6323–6328. 10.1021/acs.jpcc.6b10620.

[ref51] Basova T. V.; Kiselev V. G.; Schuster B.-E.; Peisert H.; Chassé T. Experimental and theoretical investigation of vibrational spectra of copper phthalocyanine: polarized single-crystal Raman spectra, isotope effect and DFT calculations. J. Raman Spectrosc. 2009, 40, 2080–2087. 10.1002/jrs.2375.

[ref52] Guillot N.; Shen H.; Frémaux B.; Péron O.; Rinnert E.; Toury T.; Lamy de la Chapelle M. Surface enhanced Raman scattering optimization of gold nanocylinder arrays: Influence of the localized surface plasmon resonance and excitation wavelength. Appl. Phys. Lett. 2010, 97, 023113 10.1063/1.3462068.

[ref53] Félidj N.; Aubard J.; Lévi G.; Krenn J. R.; Hohenau A.; Schider G.; Leitner A.; Aussenegg F. R. Optimized Surface-Enhanced Raman Scattering on Gold Nanoparticle Arrays. Appl. Phys. Lett. 2003, 82, 3095–3097. 10.1063/1.1571979.

[ref54] Grand J.; de la Chapelle M. L.; Bijeon J.-L.; Adam P.-M.; Vial A.; Royer P. Role of Localized Surface Plasmons in Surface-Enhanced Raman Scattering of Shape-Controlled Metallic Particles in Regular Arrays. Phys. Rev. B: Condens. Matter Mater. Phys. 2005, 72, 033407 10.1103/physrevb.72.033407.

[ref55] Li W. S.; Shen Z. X.; Feng Z. C.; Chua S. J. Temperature Dependence of Raman Scattering in Hexagonal Gallium Nitride Films. J. Appl. Phys. 2000, 87, 3332–3337. 10.1063/1.372344.

[ref56] Klemens P. G. Anharmonic Decay of Optical Phonons. Phys. Rev. 1966, 148, 845–848. 10.1103/physrev.148.845.

[ref57] Liu M. S.; Bursill L. A.; Prawer S.; Nugent K. W.; Tong Y. Z.; Zhang G. Y. Temperature Dependence of Raman Scattering in Single Crystal GaN Films. Appl. Phys. Lett. 1999, 74, 3125–3127. 10.1063/1.124083.

[ref58] Chiashi S.; Murakami Y.; Miyauchi Y.; Maruyama S. Temperature Dependence of Raman Scattering from Single-Walled Carbon Nanotubes: Undefined Radial Breathing Mode Peaks at High Temperatures. Jpn. J. Appl. Phys. 2008, 47, 2010–2015. 10.1143/jjap.47.2010.

[ref59] Keblinski P.; Cahill D. G.; Bodapati A.; Sullivan C. R.; Taton T. A. Limits of Localized Heating by Electromagnetically Excited Nanoparticles. J. Appl. Phys. 2006, 100, 054305 10.1063/1.2335783.

