# Supplementary material for: Nanoscale Thermometry of Plasmonic Structures via Raman Shifts in Copper Phthalocyanine
Source: J Phys Chem C Nanomater Interfaces. 2023 May 11;127(20):9690–8. doi: 10.1021/acs.jpcc.3c01561 (PMC10226115; doi:10.1021/acs.jpcc.3c01561)
Supplement: Supplementary file 1 — jp3c01561_si_001.pdf [file jp3c01561_si_001.pdf]

Supporting Information for

Nanoscale Thermometry of Plasmonic Structures via Raman Shifts in Copper Phthalocyanine

Pan Li[1,†], Sven H. C. Askes[1,†], Esther del Pino Rosendo[2], Freek Ariese[1], Charusheela Ramanan[1,2], Elizabeth von Hauff[1,3,4], Andrea Baldi[1,*]

[†] equal author contribution

[1] Department of Physics and Astronomy, Vrije Universiteit Amsterdam, De Boelelaan 1081, 1081 HV Amsterdam, Netherlands

[2] Max Planck Institute for Polymer Research, Ackermannweg 10, 55128 Mainz, Germany

[3] Faculty of Electrical and Computer Engineering, Technical University of Dresden, 01062 Dresden, Germany

[4] Fraunhofer Institute for Organic Electronics, Electron Beam and Plasma Technology (FEP), 01277 Dresden, Germany

Corresponding author email: a.baldi@vu.nl

Contents

S1. Focal spot size 2
S2. UV-Vis spectra 3
S3. Raman spectra 4
S4. XRD 5
S5. Absorption spectra of *β*-CuPc at varying temperatures 6
S6. Choice of proper laser power 7
Table S1. Assignment and symmetry of the Raman peaks 8
Table S2. Fitting parameters of the data in Figure 2(b) 9
S7. Sample stability during temperature calibration 10
S8. Time evolution of peak positions at various laser powers 11
S9. Extracted temperature of *β*-CuPc/Au nanodisks *vs* laser power 12
S10. Laser power dependence of bare *β*-CuPc Raman shifts 13
S11 Optical and heat-transfer modelling under Gaussian beam illumination 14
S12. Thermal transfer model 16
S13 The Raman intensity map by simulation 17
References 18

S1. Focal spot size

Plasmonic photothermal effects are strongly dependent on the laser beam profile and focal spot size [1], which can be measured by the well-known knife-edge method [2–5]. The knife-edge method is often used as a standard and precise method for Gaussian laser beam characterization. A USAF resolution test target from Thorlabs was mounted on the translation stage and moved across the beam in 0.2 μm steps while monitoring the transmitted light intensity with a power meter. To find the focal position, the intensity was measured at different Z depths. The beam widths were obtained by taking the derivative of the intensity profile at various depths and fitting it with a Gaussian function. The minimum beam width indicates the focal position. Our SERS experiments were done in a cryostat system, so we measured the laser beam width with a cryostat window above the sample, as shown in Figure S1. The full width at half maximum (FWHM) of the beam, Wy, is 2.46 um ± 0.04 μm with the cryostat window.

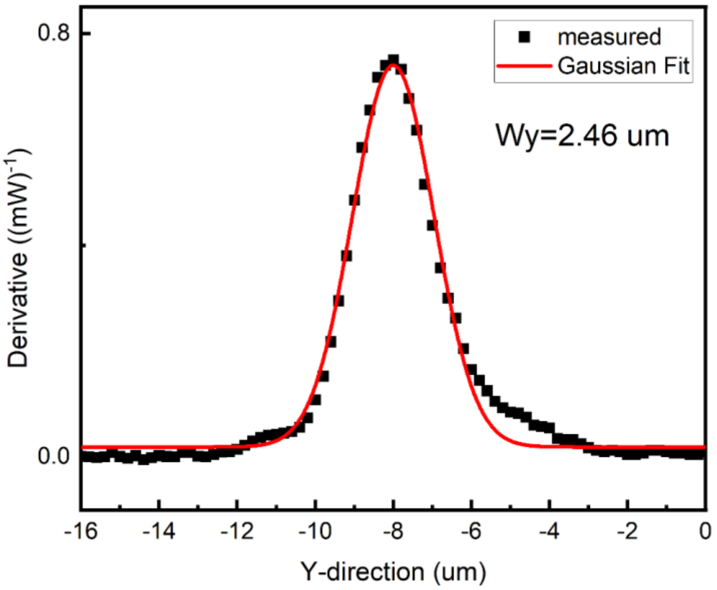

Figure S1. Laser beam profile obtained along the Y-axis of motion and fitted to a Gaussian function.

S2. UV-Vis spectra

Figure S2 shows the absorption spectra of as-deposited and annealed CuPc films, both with and without an underlying Au NP array. The absorption peaks of CuPc originate from the molecular orbitals within the aromatic system [6,7]. Three absorption peaks are observed and belong to π- π* transitions [6,7]. In the near-UV region (300-350 nm), a single peak appears and is assigned to the Soret band (or B-band). The absorption band in the visible region (600-800 nm), corresponds to the Q band and has a doublet due to Davydov splitting [6–8].

As can be seen, the position and the relative intensity of the Q-bands are different for as-deposited (black line) and annealed (red line) CuPc films on a glass substrate. The band red-shifts upon annealing because of the structural modification. For as-deposited CuPc thin film, the intensity of the higher energy peak (at 619 nm) is larger than that of the lower energy peak (at 694 nm), as expected for α-CuPc [6–8]. For annealed CuPc thin film, this intensity ratio is inverted, indicating the formation of *β*-CuPc [7,8].

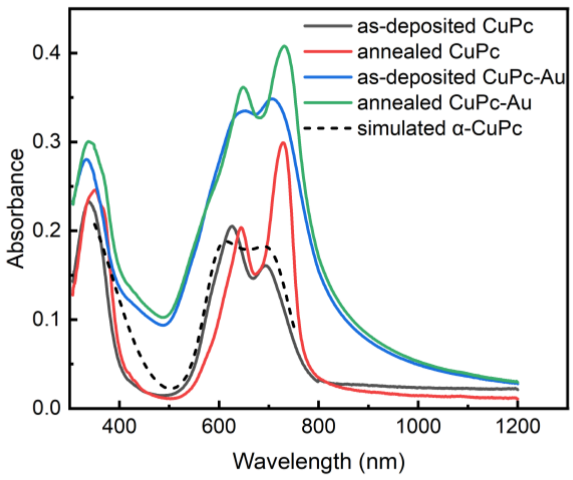

Figure S2. The UV-Vis spectra of as-deposited 20 nm CuPc film (black line) on bare glass and the same film annealed at 300°C in N_2 for 3 h (red line), and the as-deposited nearly 20 nm CuPc film on Au nanodisks (blue line) and the same film annealed at 300°C in N_2 for 3 h (green line). The black dashed line is the calculated extinction spectrum of 20 nm thick α-CuPc on glass.

We also studied the absorption spectrum of a 20 nm thick CuPc film as-deposited on a Au nanodisk array (blue line) and after annealing (green line). A similar red-shift and intensity change is observed upon annealing, indicating the formation of the *β*-phase.

S3. Raman spectra

Previous investigations of the Raman tensors and polarized Raman spectra of oriented CuPc single crystals have shown that the low wavenumber region (< 200 cm^{-1}) results from the lattice vibrations and is defined according to the crystal symmetry C_{2h} [9], whereas the high wavenumber region (> 200 cm^{-1}) corresponds to intramolecular modes and is defined following the molecular symmetry D_{4h} [9]. Figure S3 plots the Raman spectra of as-deposited and annealed CuPc films with 60 nm thickness measured at room temperature. The annealed film (red line) shows additional peaks at 850 cm^{-1} and 154 cm^{-1} (indicated with *), confirming the formation of the β-phase [10,11].

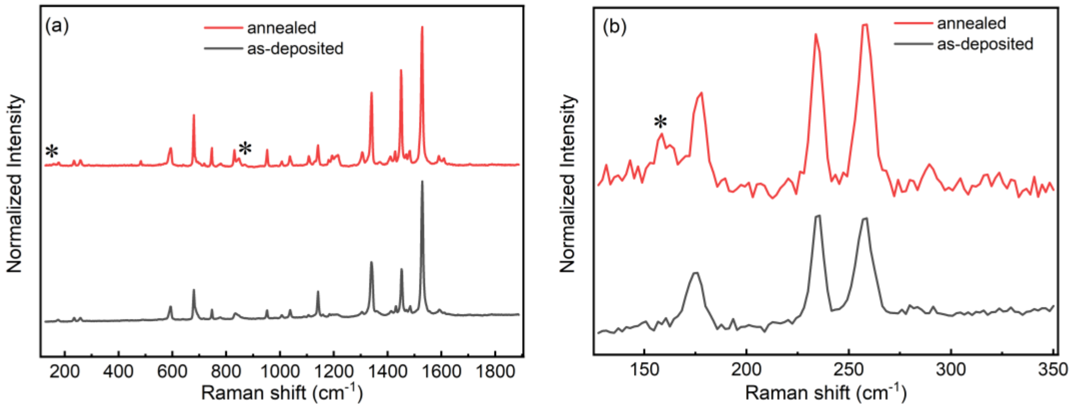

Figure S3: (a) Raman spectra of a 60 nm thick CuPc film both as-deposited and annealed at 300 °C in N_2 for . (b) Zoom-in on the low wavenumber region; excitation = 532 nm.

S4. XRD

Figure S4 shows the XRD pattern measured over a 300 nm thick CuPc film evaporated onto a glass substrate and annealed at 300 °C. The peaks at 6.96° and 9.16° can be attributed to β-CuPc [10,12,13].

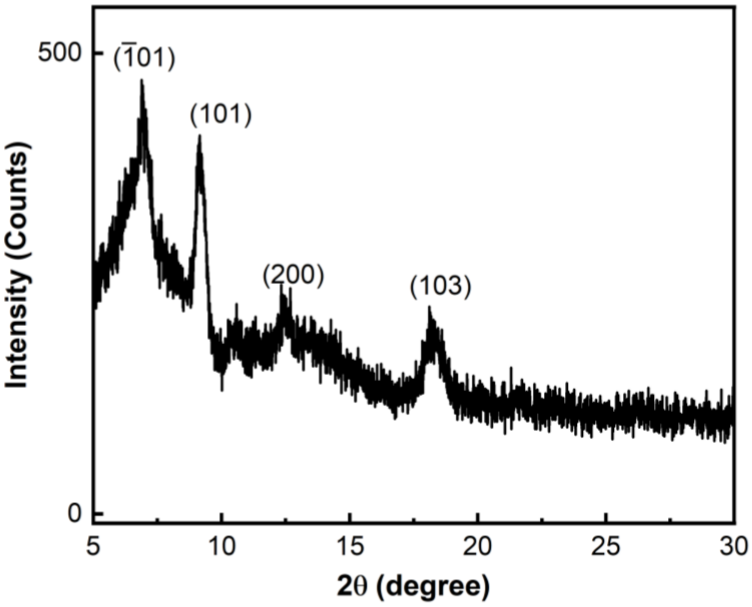

Figure S4 The XRD patterns of a 300 nm thick annealed CuPc film on a glass substrate.

S5. Absorption spectra of β-CuPc at varying temperatures

The thermal stability of β-CuPc films was tested via temperature-dependent UV-Vis spectroscopy. As shown in Figure S5, the spectral position and line shape of the peaks do not change, suggesting that the sample is not undergoing any phase change in the 300-495 K temperature range (see also Fig S2).

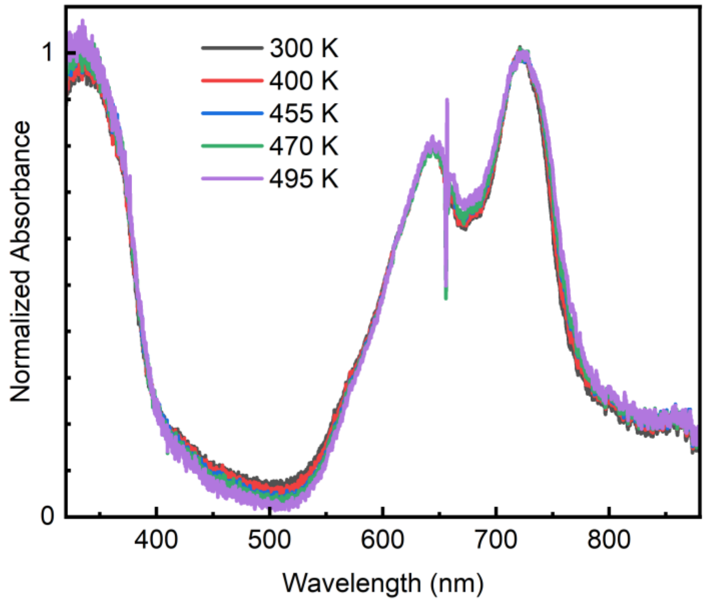

Figure S5. UV-Vis absorption spectra of a 60 nm thick β-CuPc film on bare glass, measured from 300 K to 495 K in vacuum in ascending order with a heating rate of ~20 K/min.

S6. Choice of proper laser power

As a thermometer, the calibration would be done for *β*-CuPc film on Au nanodisks by temperature dependent Raman measurements. During the calibration process, any fluctuation in the thermal environment could introduce extra errors. Firstly, we considered the local heating effect from the laser by the photon absorption and the accumulated irradiation time. Therefore, we compared the influence of different laser powers and irradiation times on the Raman peak shift. Figure S6(a) shows the measured Raman spectra of *β*-CuPc thin films on Au nanodisks under 0.045 mW and 0.2 mW laser power with an irradiation time of 80 s and under 0.5 mW with an irradiation time of 40 s. Increasing the laser power from 0.045 mW to 0.2 mW significantly improved the signal-to-noise ratio of the Raman peak without giving rise to significant heating, as indicated by the small shift in peak position (~0.25 cm^{-1}). Increasing the laser power even further did not improve the S/N, possibly due to the effect of laser-induced heating of the film.

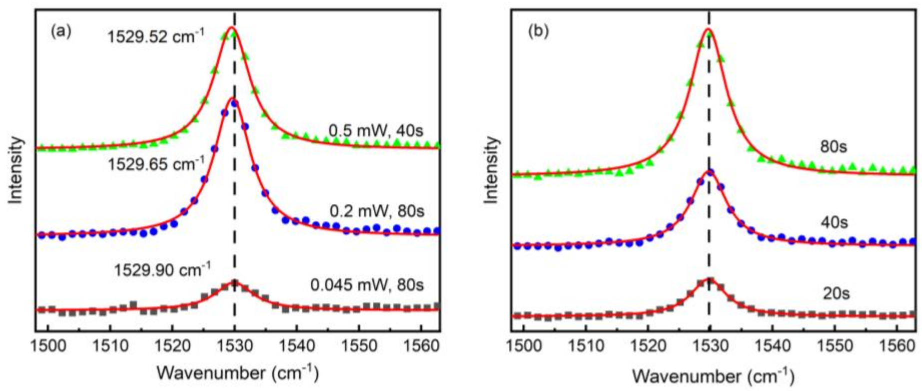

Figure S6. (a) Raman spectra of *β*-CuPc film on Au nanodisks at the peak position of 1530 cm^{-1} with different laser powers; (b) Raman spectra of *β*-CuPc film on Au nanodisks at the peak position of 1530 cm^{-1} with different irradiation times at a fixed laser power of 0.2 mW. The red lines represent the Lorentz fitting curves.

Under a laser power of 0.2 mW, our Au nanodisk array generates a temperature increase of ~6 K, as calculated from equations (6)-(8) in the main text. Such a temperature increase is very small compared to the temperature range explored in this study (100 K – 500 K). Figure S6(b) shows the same Raman peaks measured under different laser irradiation times with a fixed laser power of 0.2 mW, indicating no significant peak shift with increasing irradiation time.

Given the above considerations, the temperature dependent Raman spectra of *β*-CuPc films were measured with a laser power of 0.2 mW and irradiation times of 80 s.

Table S1. Assignment and symmetry of the Raman peaks

Wavenumber	Symmetry	Assignment
**1530 cm^{-1}**	B_{1g}	C_α–N_β
**1453 cm^{-1}**	B_{1g}	C_β–C_β, C_β–C_γ–H
	B_{2g}	C_α–N_β, N_α–C_α–C_β, C–C–H
**1341 cm^{-1}**	B_{1g}	C_β –C_β, C_α–C_β–C_β, C_γ–C_δ, C_β–C_γ

The vibrational modes for the CuPc molecule are given by [9,12] $\Gamma_{vib.} = 14A_{1g} + 13A_{2g} + 14B_{1g} + 14B_{2g} + 13E_g + 6A_{1u} + 8A_{2u} + 7B_{1u} + 7B_{2u} + 28E_u$. The A_{1g}, B_{1g}, B_{2g} and E_g modes are Raman allowed, with A_{1g}, B_{1g}, B_{2g} corresponding to in-plane vibrations and E_g representing an out-of-plane one. The A_{2u} and E_u modes are IR active. As shown in Figure 1(a) of the main text, in the case of B_{1g} type vibrations, the displacements of atoms are symmetric along the two-fold C_2' axis passing through the central copper atom and N_α atoms. For the B_{2g} vibrational modes, the atom displacements are symmetric along the C_2'' axis through the central copper atom and N_β atoms.

Table S2. Fitting parameters of the data in Figure 2(b)

Below are the parameters used to fit the three trends in Figure 2(b) using equations (1) and (2) in the main text.

Peak	ω_0	A	B
1530 cm^{-1}	1532.73±0.08 (cm^{-1})	10.46±0.99 (cm^{-1})	0.21±0.02
1453 cm^{-1}	1454.50±0.04 (cm^{-1})	10.51±2.19 (cm^{-1})	0.34±0.05
1341 cm^{-1}	1342.53±0.07 (cm^{-1})	9.26 ± 1.50 (cm^{-1})	0.31±0.04

S7. Sample stability during temperature calibration

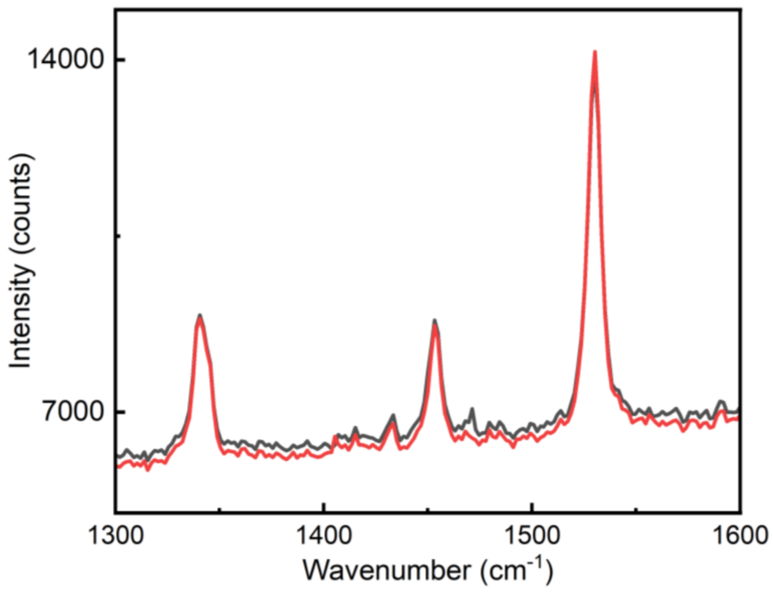

Figure S7. Raman spectra of *β*-CuPc on Au nanodisks measured at 296 K before (black) and after (red) heating to 500 K.

S8. Time evolution of peak positions at various laser powers

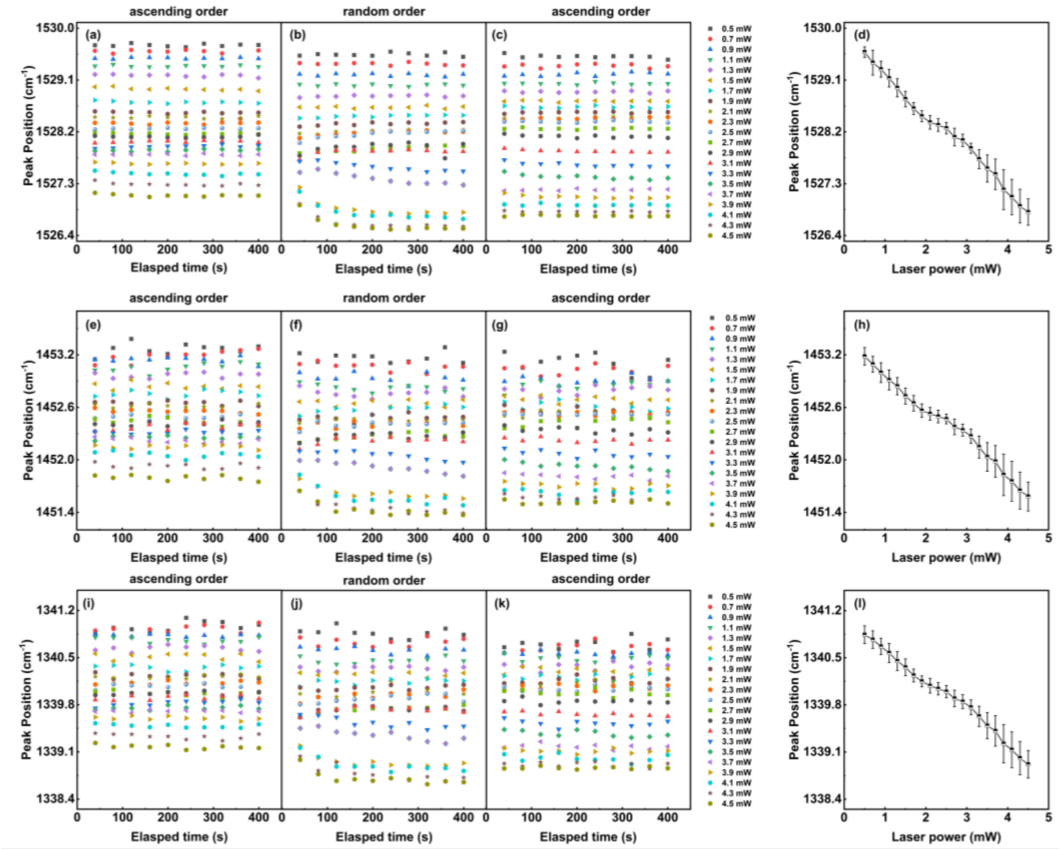

Figure S8. (a-c) present the 1530 cm^{-1} peak positions of a β-CuPc on Au nanodisks sample as a function of the elapsed time. The measurements were repeated on three different days, with laser power changing in ascending order, random order, and ascending order, respectively. There was 15 min interval between two laser power measurements. 10 spectra were measured for each laser power. Each spectrum was acquired with exposure time of 10 s and 4 accumulations. The peak positions are obtained by fitting with Lorentzian functions. (d) Peak position averaged from the 30 measurements in (a-c) as a function of laser power. The error bar is the standard deviation. (e-h) and (i-l) are the same as (a-d), but for the 1453 cm^{-1} and 1341 cm^{-1} peaks, respectively.

S9. Extracted temperature of *β*-CuPc/Au nanodisks *vs* laser power

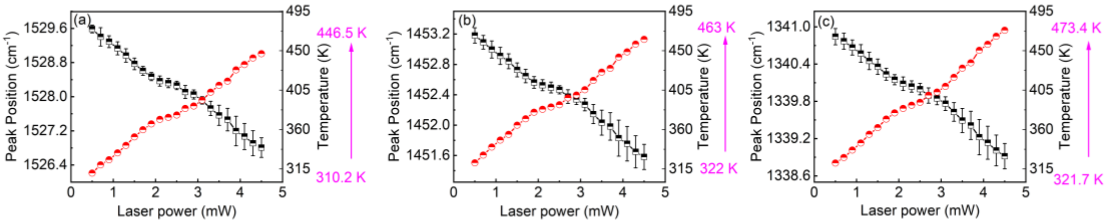

Figure S9. (a)-(c) Peak positions (black dots) and extracted temperatures (red dots) of a *β*-CuPc/Au nanodisks sample, shown as a function of increasing laser power. The extracted temperatures of the three peaks range from 310.2 K to 446.5 K, 322 K to 463 K, and from 321.7 K to 473.4 K, corresponding to the laser power changing from the lowest value to highest value.

S10. Laser power dependence of bare *β*-CuPc Raman shifts

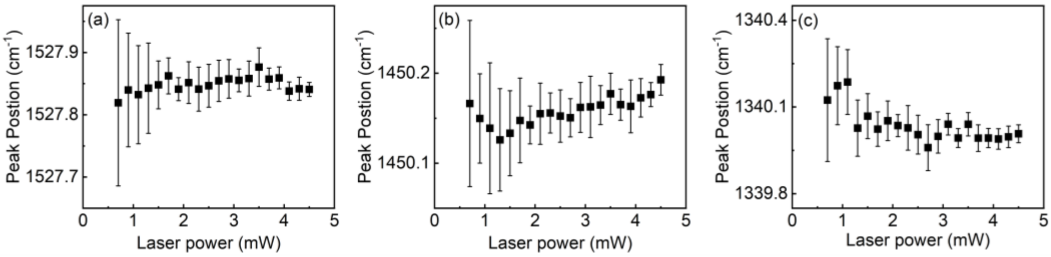

Figure S10. Laser power dependence of the Raman shift of the three dominant peaks for a 20 nm thick *β*-CuPc film on glass. Each data point is the average of 10 consecutive acquisitions at each laser power. Every spectrum was obtained with an exposure time of 10 s and 8 accumulations.

S11 Optical and heat-transfer modelling under Gaussian beam illumination

The spatial electric-field enhancement (*E*(x,y,z)) and spatial heating profile (*q(x,y,z)*) of Au-CuPc arrays was modelled as follows. First, finite difference time domain (FDTD) simulations were conducted using Lumerical software to solve Maxwell's equations for the nanoparticle geometry, under the experimentally determined 532 nm Gaussian beam illumination (2.4 µm FWHM; 2.05 µm 1/e^2 beam waist radius, see Figure S1). The Au nanoparticles consisted of 90 nm diameter cylinders of 30 nm height, of which the bottom 3 nm consisted of a Cr adhesion layer. Because the CuPc was deposited by evaporation, the substrate was covered by a uniform 21 nm CuPc layer and the Au nanoparticles were covered by 21 nm thick cylinders (with 90 nm diameter). The simulated part of the square array consisted of a 32 × 32 nanoparticle sub-lattice (5.12 µm × 5.12 µm) on glass and x&y symmetry was applied to minimize computational requirements. For Au, the built-in optical constants from Johnson and Christy were used [14]. For CuPc, optical constants were taken from Singh and Ravindra [15]. The background refractive index was set to 1.000 (air/vacuum).

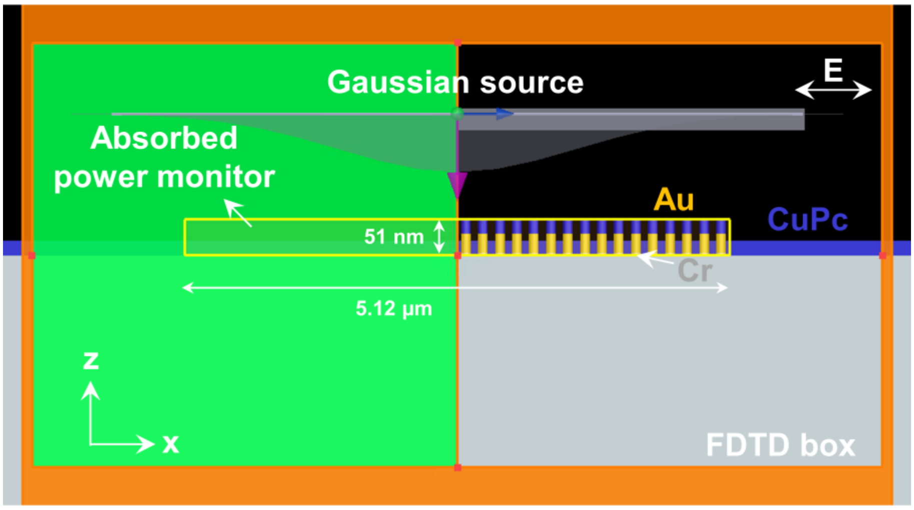

Figure S11. Screenshot of the Lumerical FDTD simulation space used for Gaussian beam simulations.

An advanced absorbed power monitor was used to retrieve both the three-dimensional electric field, $E(x, y, z)$, and the absorbed power profile within the nanoparticle, $P_{abs}(x, y, z)$, which is proportional to the product of the electric field intensity, $|E(x, y, z)|^2$, at light frequency ω, and the imaginary part of the permittivity, ε'':

$$P_{abs}(x, y, z) = -0.5\omega|E(x, y, z)|^2\varepsilon'' \quad (1)$$

To normalize the absorption profile, all output values were divided by the global average within the optically heated domain (Au + Cr + CuPc containing voxels) to yield $\bar{P}_{\text{abs}}(x,y,z)$, which was subsequently imported in COMSOL as an interpolated function. Finally, the spatial heating function $q(x,y,z)$ was obtained by multiplying $\bar{P}_{\text{abs}}(x,y,z)$ by the laser power (P in W), the experimentally measured extinction ($I_{\text{ext}} = 29\%$), and the fraction of extinguished light that is absorbed, as calculated from FDTD simulations ($f_{\text{abs}} = \frac{\sigma_{\text{abs}}}{\sigma_{\text{abs}}+\sigma_{\text{sca}}} = 0.85$), and dividing by 4 to account for the four-fold geometrical symmetry in the simulation:

$$q(x,y,z) = \frac{1}{4}\, P\, I_{\text{ext}}\, f_{\text{abs}}\, \bar{P}_{\text{abs}}(x,y,z) \qquad (2)$$

COMSOL Multiphysics was used to solve the differential equation for conductive heat transfer (Equation 3), where ρ is the material density (kg/m^3), C_p is the heat capacity under constant pressure (J/kg.K), T is the temperature (K) and κ is the thermal conductivity (W/m.K).

$$\rho C_p \frac{\partial T}{\partial t} = \nabla[\kappa \nabla T] + q(x,y,z) \qquad (3)$$

S12. Thermal transfer model

Heat transfer through convection was neglected as the experiments were carried out in (partial) vacuum, i.e. the vacuum was modelled with 0.1% of the thermal conductivity of air. A cubic geometry was designed of 10×10×10 µm with the 32×32 particle Au-CuPc array at the center, on glass substrate and in partial vacuum (1 mbar), see Figure S12a. The array domain was finely meshed, while the outer domain was coarsely meshed (~ 3×10^6 domain elements in total). A four-fold symmetry was used to minimize computational requirement, with symmetry boundary conditions on the inner boundaries; Thus, the actual simulation space was 20×20×10 µm (xyz). The outer boundary temperature was fixed at 293.15 K, assuming a negligible temperature gradient beyond this volume in the Raman microscopy experiment. Built-in heat capacities and thermal conductivities were used for glass (clear soda lime silica), chromium, and gold. For CuPc we used an estimated heat capacity of 1200 J/kg.K (a reasonable value for organic semiconductors) [16] and a thermal conductivity of 0.39 W/m.K [17]. A direct and fully coupled stationary solver was used to calculate a steady-state solution at each experimental laser power.

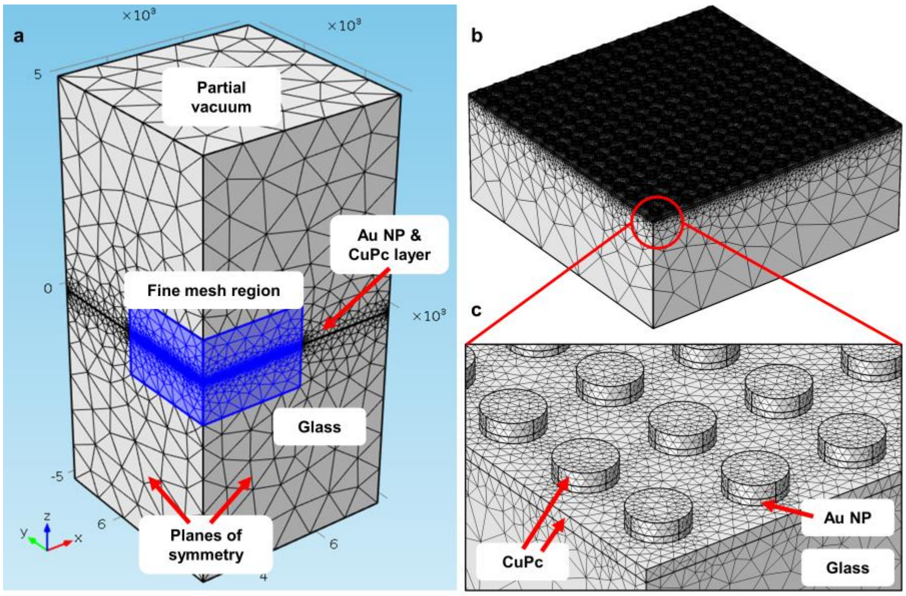

Figure S12. COMSOL simulation geometry and simulation mesh for Au nanodisks embedded in CuPc. a) Complete geometry and meshing. b&c) Detailed geometry and mesh of the finely meshed region with an array of 16 × 16 nanoparticles shown (blue in panel a); the 30 nm tall Au nanostructures are almost completely embedded in deposited CuPc.

S13 The Raman intensity map by simulation

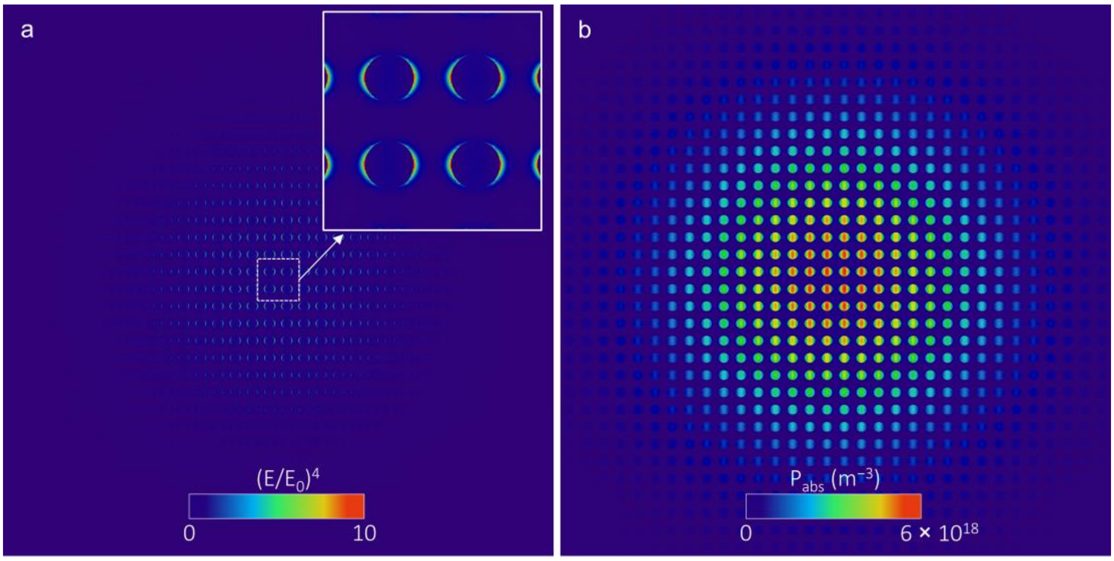

Figure S13. Raman intensity map $(E/E_0)^4$ (a) and absorbed power (b) of a 32 × 32 nanoparticle array (5.12 × 5.12 µm area) under 2.4 µm FWHM Gaussian beam illumination at z = 15 nm.

References

(1)Baffou, G.; Berto, P.; Bermúdez Ureña, E.; Quidant, R.; Monneret, S.; Polleux, J.; Rigneault, H. Photoinduced Heating of Nanoparticle Arrays. *ACS Nano* **2013**, *7* (8), 6478–6488.

(2)Chen, Q.; Chen, Q.; Qi, H.; Ruan, L.; Ren, Y. Experimental Comparison of Photothermal Conversion Efficiency of Gold Nanotriangle and Nanorod in Laser Induced Thermal Therapy. *Nanomaterials* **2017**, *7* (12), 416.

(3)Bonnett Del Alamo, M.; Soncco, C.; Helaconde, R.; Bazo Alba, J. L.; Gago, A. M. Laser Spot Measurement Using Simple Devices. *AIP Advances* **2021**, *11* (7), 075016.

(4)de Araújo, M. A.; Silva, R.; de Lima, E.; Pereira, D. P.; de Oliveira, P. C. Measurement of Gaussian Laser Beam Radius Using the Knife-Edge Technique: Improvement on Data Analysis. *Appl. Opt.* **2009**, *48* (2), 393.

(5)Zada, L.; Fokker, B.; Leslie, H. A.; Vethaak, A. D.; de Boer, J. F.; Ariese, F. Stimulated Raman Scattering Simulation for Imaging Optimization. *J. Eur. Opt. Soc.-Rapid Publ.* **2021**, *17* (1), 10.

(6)Karan, S.; Basak, D.; Mallik, B. Copper Phthalocyanine Nanoparticles and Nanoflowers. *Chemical Physics Letters* **2007**, *434* (4–6), 265–270.

(7)Karan, S.; Mallik, B. Effects of Annealing on the Morphology and Optical Property of Copper (II) Phthalocyanine Nanostructured Thin Films. *Solid State Communications* **2007**, *143* (6–7), 289–294.

(8)Tong, W. Y.; Chen, H. Y.; Djurišić, A. B.; Ng, A. M. C.; Wang, H.; Gwo, S.; Chan, W. K. Infrared Photoluminescence from α- and β-Copper Phthalocyanine Nanostructures. *Optical Materials* **2010**, *32* (9), 924–927.

(9)Basova, T. V.; Kiselev, V. G.; Schuster, B.-E.; Peisert, H.; ChassÃ©, T. Experimental and Theoretical Investigation of Vibrational Spectra of Copper Phthalocyanine: Polarized Single-Crystal Raman Spectra, Isotope Effect and DFT Calculations. *J. Raman Spectrosc.* **2009**, *40* (12), 2080–2087.

(10)Zou, T.; Wang, X.; Ju, H.; Zhao, L.; Guo, T.; Wu, W.; Wang, H. Controllable Molecular Packing Motif and Overlap Type in Organic Nanomaterials for Advanced Optical Properties. *Crystals* **2018**, *8* (1), 22.

(11)Kolesov, B. A.; Basova, T. V.; Igumenov, I. K. Determination of the Orientation of CuPc Film by Raman Spectroscopy. *Thin Solid Films* **1997**, *304* (1–2), 166–169.

(12)Ghorai, U. K.; Mazumder, N.; Mamgain, H.; Roy, R.; Saha, S.; Chattopadhyay, K. K. Raman Spectroscopic Observation of Gradual Polymorphic Transition and Phonon Modes in CuPc Nanorod. *J. Phys. Chem. C* **2017**, *121* (11), 6323–6328.

(13)Prabakaran, R.; Kesavamoorthy, R.; Reddy, G. L. N.; Xavier, F. P. Structural Investigation of Copper Phthalocyanine Thin Films Using X-Ray Diffraction, Raman

Scattering and Optical Absorption Measurements. *Phys. Status Solidi A* **2002**, *229* (3), 1175–1186.
(14)Johnson, P. B.; Christy, R. W. Optical Constants of the Noble Metals. *Phys. Rev. B* **1972**, *6* (12), 4370–4379.
(15)Singh, P.; Ravindra, N. M. Optical Properties of Metal Phthalocyanines. *J Mater Sci* **2010**, *45* (15), 4013–4020.
(16)Duda, J. C.; Hopkins, P. E.; Shen, Y.; Gupta, M. C. Thermal Transport in Organic Semiconducting Polymers. *Appl. Phys. Lett.* **2013**, *102* (25), 251912.
(17)Jin, Y.; Shao, C.; Kieffer, J.; Pipe, K. P.; Shtein, M. Origins of Thermal Boundary Conductance of Interfaces Involving Organic Semiconductors. *Journal of Applied Physics* **2012**, *112* (9), 093503.
